# Medial prefrontal cortex is selectively involved in response selection using visual context in the background

**DOI:** 10.1101/lm.025890.112

**Published:** 2012-06

**Authors:** Inah Lee, Ji Yun Shin

**Affiliations:** Department of Brain and Cognitive Sciences, Seoul National University, Gwanak-ro, Gwanak-gu, Seoul 151-742, Korea

## Abstract

The exact roles of the medial prefrontal cortex (mPFC) in conditional choice behavior are unknown and a visual contextual response selection task was used for examining the issue. Inactivation of the mPFC severely disrupted performance in the task. mPFC inactivations, however, did not disrupt the capability of perceptual discrimination for visual stimuli. Normal response selection was also observed when nonvisual cues were used as conditional stimuli. The results strongly suggest that the mPFC is not necessarily involved in the inhibition of response or flexible response selection in general, but is rather critical when response selection is required conditionally using visual context in the background.

“Contextual response selection” enables an organism to respond flexibly and adaptively in various situations when encountering similar objects (potentially associated with conflicting responses) across different contextual settings. It is implicated in the literature that the prefrontal cortex (PFC) is critically involved in making flexible responses across different contexts (Kesner and Ragozzino 2003; Haddon and Killcross 2006; Marquis et al. 2007; Lee and Solivan 2008; Horga et al. 2011). The PFC receives direct afferent fibers from the hippocampus (Jay and Witter 1991; Thierry et al. 2000), which is one of the critical structures for contextual memory tasks (Hirsh 1974; Good and Honey 1991; Kim and Fanselow 1992; Kim and Lee 2011). Presumably related to such “contextual connections,” perturbations of PFC lead to severe impairment in contextual response selection tasks in which visual contextual cues play significant roles (Haddon and Killcross 2006; Jo et al. 2007; Marquis et al. 2007; Lee and Solivan 2008).

In prior studies, however, behavioral tasks required subjects to learn complicated multiple relationships between noncontextual stimuli (e.g., object, light, tone, etc.) and visual contexts (e.g., room cues) when testing contextual response selection (Haddon and Killcross 2006; Marquis et al. 2007; Lee and Solivan 2008). In other cases, rats learned biconditional associations between two different objects and locations in a maze and flexibly responded to a context-relevant object for obtaining reward (Lee and Solivan 2008). What these studies have tested is whether rats could learn associations between multiple individual cues and discrete contexts and whether PFC was necessary in flexibly responding (or not responding) to particular cue-context paired associates. It is unclear in these behavioral paradigms, however, how much “response selection” component was tested and also what proportion of resulting performance deficits following PFC manipulations could be attributed to contextual response selection per se. In the current study, in order to establish a more straightforward relationship between context and response selection behavior, we removed the requirement of associating other sensory cues (e.g., light, tone, and objects) with context and just made a single object (i.e., sand-filled jar) allow two distinctively different types of motor responses (digging the sand in the jar or pushing the jar).

Rats (*n* = 8, male Long-Evans) were trained in a visual contextual response selection (VCRS) task (Fig. 1A,B) and were implanted with bilateral cannulae (26G) targeting the mPFC (2.7 mm anterior to bregma, 1.5 mm lateral to midline at a 10° angle, 4.9 mm ventral to the skull surface) (Fig. 1C) once they reached performance criterion (≥75% correct choices for both contexts for two consecutive days). In the VCRS task (32 trials/session), the rat was required to push a sand-filled jar (480 g) when an array of peripheral LCD panels (each panel, 17″ TFT LCD monitor) displayed a certain visual context (e.g., pebbles pattern) (Fig. 1A), but the rat must dig the sand in the same object when another visual context (e.g., zebra pattern) (Fig. 1B) was shown in order to obtain the reward (cereal). The stimulus–response relationships associated with rewards were counterbalanced among the rats. The jar was placed in the center of the surrounding LCD panels in the background. We prevented rats from using a potential olfactory strategy by mixing sand with ground Cheerios powder (1:5 ratio). Rats were unable to tell whether a piece of cereal was in the sand or not by smelling it because they showed digging behavior even when the reward was missing in some probe trials. Since the jar was heavy and the cereal reward was buried deep in the jar, the two response modes were equally effortful behaviors in which the rat should be intentionally engaged for obtaining reward, and thus were easily discriminated by the experimenter in the testing room. If the rat moved the jar (even a little without exposing the food well underneath it), it was considered a pushing response. Once the rat touched the sand with both front paws, it was considered a digging response. No correction was allowed once a wrong behavioral selection was made. One of the rats was dropped from further analysis due to misplacement of cannula tips. For the postsurgical testing of performance, phosphate-buffered saline (SAL, 0.5 µL per site) was injected on day 1 and muscimol (MUS, 0.5 µg/0.5 µL per site), a GABA-A receptor agonist, was injected afterward for 2 d in a row. SAL was injected again on the last day for checking the baseline performance. All cannula tip positions of the remaining seven rats were located in the mPFC (Fig. 1C). The diffusion range of MUS was estimated using fluorescent MUS after all experiments were finished (see Jo and Lee 2010 for detailed methods) and the results showed that MUS diffusion covered mostly the prelimbic and infralimbic cortices (spanning anteroposteriorly +3.5 to +1.9 mm from bregma and ±1.2 mm mediolaterally) on average. Rats injected with SAL in the mPFC before and after MUS injections were normal in performing the VCRS task (Fig. 1D). The same rats, however, were severely impaired when MUS was injected in the mPFC compared with SAL conditions (Fig. 1D). A repeated-measures ANOVA showed a highly significant effect of drug condition (*F*_(3,21)_ = 20.3, *P* < 0.0001). Post hoc comparisons (Tukey-Kramer) revealed significant differences between all SAL and MUS conditions (*P-*values of <0.05). However, despite the seemingly increased performance in the second MUS condition (Fig. 1D), no statistical significance was found between the first and second MUS conditions. No significant difference in performance was found between SAL conditions. No significant effect of drug was found when average response latency (from start-box exit to response) was compared across days, suggesting that MUS did not disrupt generic sensory-motor capabilities. The results strongly demonstrate that the mPFC is critical for visual contextual response selection.

**Figure 1.**
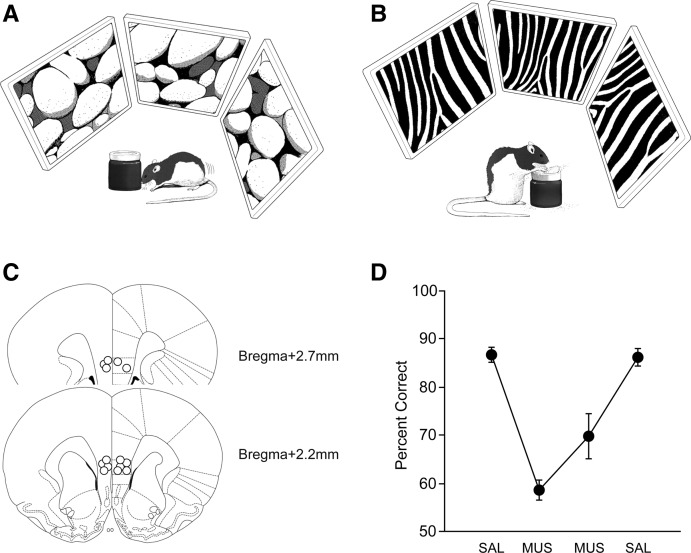
Visual contextual response selection (VCRS) task. (*A*) Cartoon version of contextual behavioral responses (*A*, pushing in pebbles context; *B*, digging in zebra context) associated with the target object (sand-filled jar). (*C*) Schematic illustration of cannula-tip positions in the mPFCs of all of the rats used in behavioral data analysis. (*D*) Performance in the VCRS task with SAL and MUS injections in the mPFC (50% = chance-level performance). Note the significant decreases in performance with mPFC inactivations with MUS as compared with SAL conditions. (Mean ± SEM.)

In the VCRS task, the rat must first identify the surrounding visual context presented in a given trial in order to choose a correct response associated with the context. We examined whether the capability of visual discrimination was impaired in the mPFC-inactivated rats using a T-maze in a visual cue discrimination (VCD) task (Fig. 2A). In the VCD task (32 trials/session), the rat was simply required to turn to one of the arms associated with the peripheral LCD monitor displaying the rewarding visual stimulus and to displace a metal washer to retrieve a cereal reward in the food well. Since the motor behavioral requirement (i.e., turning response) was identical for both arms, the VCD task significantly reduced the response-conflict component (i.e., digging vs. pushing) in the VCRS task and allowed testing for the capability of perceptual discrimination of visual stimuli in a more straightforward manner. When the same rats from the VCRS task were trained (criterion = ≥75% correct choices for two consecutive days; reward contingencies counterbalanced among rats) and tested in the VCD task using the within-subjects design (i.e., SAL-MUS-MUS-SAL as in VCRS), rats exhibited >70% correct performances in all SAL and MUS conditions (Fig. 2B). No significant differences were found statistically between SAL and MUS conditions (*F*_(3,21)_ = 1.06, *n.s.*; repeated-measures ANOVA). The results suggest that the mPFC is not required for discriminating complex visual stimuli per se, and the deficits observed in the VCRS task are likely attributable to the impairment in retrieving the association between the representation of the visual context and its paired behavioral response.

**Figure 2.**
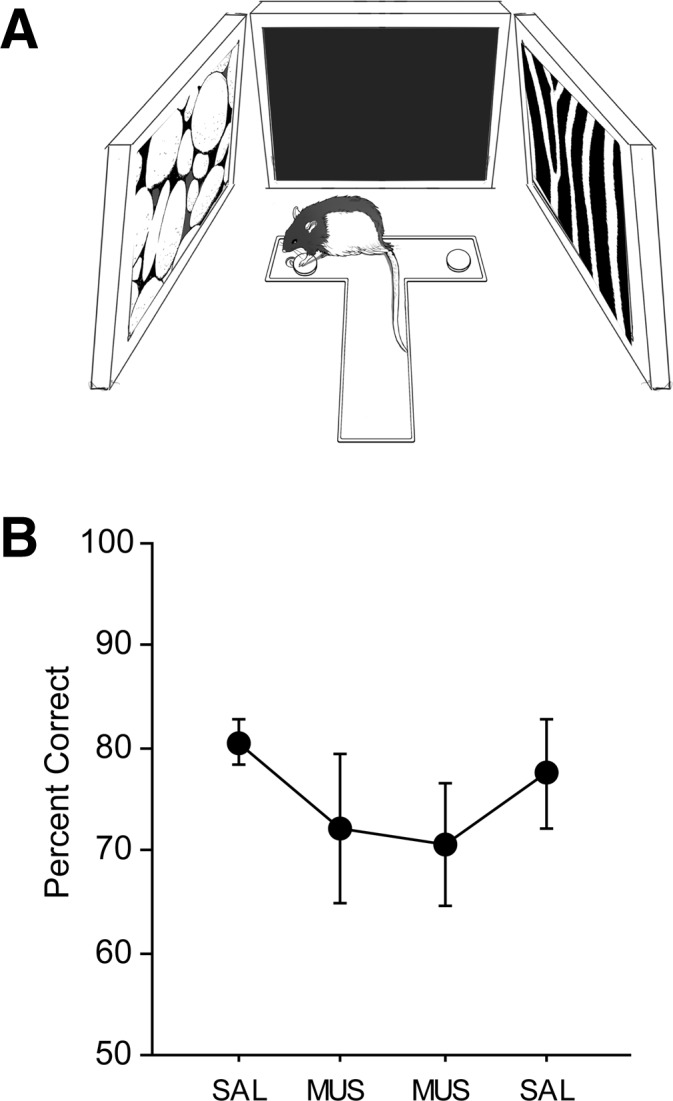
Visual cue discrimination (VCD) task. (*A*) Behavioral paradigm. The rat was rewarded when displacing the washer in the arm in front of the LCD monitor that showed a rewarding visual stimulus. (*B*) Performance in the VCD task with SAL and MUS infusions in the mPFC. No significant impairment was observed in performance with mPFC inactivation compared with SAL conditions. (Mean ± SEM.)

Another possibility, however, is that the mPFC is responsible for driving the motor system for selective activation of a certain behavioral circuit (e.g., pushing vs. digging). In order to test this possibility, we used a tactile-cued response selection (TCRS) task (32 trials/session) in which tactile cues (wire mesh or soft shelf liner) were used for response selection (Fig. 3A). Once a trial started, the rat exited the start box and entered the response platform. The animal was able to sample the tactile cue on the floor as it entered the platform until it responded to the sand-filled jar with either a digging or pushing response (depending on the tactile cue). The relationships between tactile stimuli and dig–push responses were counterbalanced among rats. The same within-subject drug-injection schedule (SAL-MUS-MUS-SAL) used for prior tasks was used. The rats injected with MUS in the mPFC performed as well (>80%) as in SAL conditions and showed normal tactile-cued responses to the sand-filled jar (Fig. 3B). No significant drug effect was observed (*F*_(3,21)_ = 2.75, *n.s.*; repeated-measures ANOVA).

**Figure 3.**
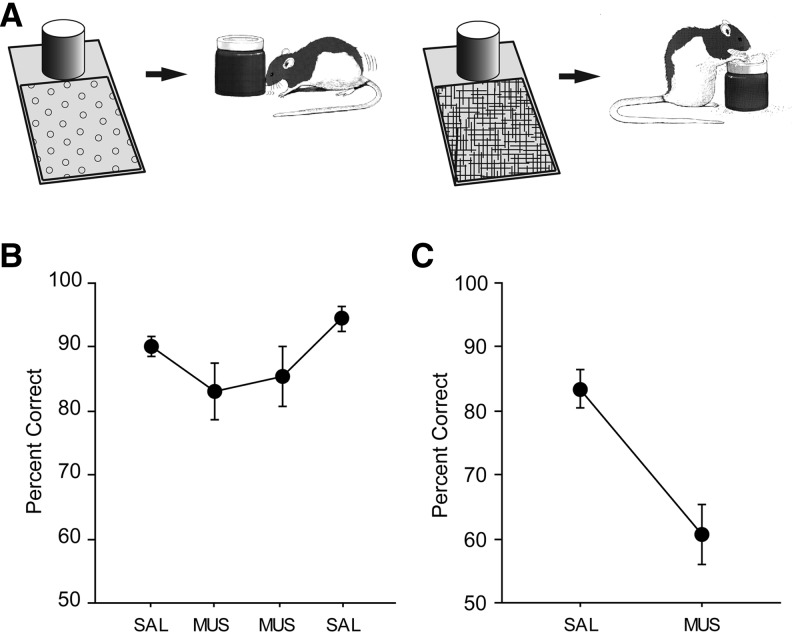
Tactile-cued response selection (TCRS) task (*A*,*B*) and VCRS task (*C*). (*A*) Behavioral paradigm. A floor insert with tactile cue (*left*, soft shelf liner; *right*, wire mesh) was used for informing which response (pushing or digging) was appropriate toward the same object (sand-filled jar) for obtaining a reward in a given trial. (*B*) Rats showed no significant differences in performance between SAL and MUS conditions. (Mean ± SEM.) (*C*) Performance in the VCRS task run after the TCRS task in the same rats. The rats remembered the VCRS task well with SAL injections in the mPFC, whereas performance was severely affected again by MUS injections. (Mean ± SEM.)

One may reason that the null effects of MUS in the mPFC in both VCD and TCRS tasks might be related to a possibility that MUS could have exerted less inhibitory influence on the mPFC in later days of testing as the system was exposed to MUS for multiple days throughout the study. This is unlikely because, when tested in the VCRS task after the completion of the TCRS task, MUS again severely disrupted performance in the same animals, whereas no such impairment was observed with SAL (Fig. 3C) (*F*_(1,7)_ = 15.39, *P* < 0.01, repeated-measures ANOVA). The results overall strongly suggest that mPFC is selectively involved in contextual response selection involving distal visual cues in the background.

The functional involvement of mPFC in response selection was apparently limited to the VCRS task in which the contextual stimulus served as a critical conditional cue implicitly from the background at the time of conditional response selection. The background visual stimuli in the VCRS task worked as contextual cues implicitly or indirectly at the time of response selection as compared with the explicit elemental cues in both VCD and TCRS tasks. Hirsh (1974) emphasized this implicit nature of influence by background stimuli when defining contextual learning and memory in the hippocampus. Although the current study manipulated mPFC instead of the hippocampus, the results connect well to such contextual theory. Alternatively, one may focus on the difference between distal and local cues to explain different results between VCRS and TCRS tasks. However, the mPFC-inactivated rats were normal in the VCD task where distal cues were also used as in the VCRS task. Therefore, the impairment in the VCRS task may not be simply attributable to the distal nature of visual stimulus. Despite the difference in the type of behavioral responses between VCRS and VCD tasks (i.e., different behavioral interactions with the same object vs. simple orienting response toward a visual cue), complexity in motor response per se may not be a critical factor underlying the performance deficits with mPFC inactivations, because the TCRS task was identical to the VCRS task with respect to the required behavioral responses. The results from the TCRS task strongly suggest that some of the key cognitive components (including resolving conflicts between different behavioral responses) for conditionally choosing a proper response toward the same object were intact without the mPFC. Literature also suggests that mPFC-inactivated rats are normal in inhibiting inappropriate responses and flexibly choosing correct ones in certain situations (Bussey et al. 1997; Delatour and Gisquet-Verrier 1999; Chudasama et al. 2003; Ragozzino et al. 2003; McDonald et al. 2007; Floresco et al. 2008; Hayton et al. 2010).

Attention related to task difficulty may also explain the differential influences of MUS in the mPFC in different tasks in our study. Specifically, several hypotheses have been proposed for explaining mPFC functions, including working memory (Goldman-Rakic 1990; Granon et al. 1994; Delatour and Gisquet-Verrier 1996), response selection (Delatour and Gisquet-Verrier 1996; Haddon and Killcross 2006; Marquis et al. 2007), high-level attention or effortful processing (Granon et al. 1995; Delatour and Gisquet-Verrier 1996; Muir et al. 1996; Bussey et al. 1997), response inhibition (Chudasama et al. 2003; McDonald et al. 2007; Hayton et al. 2010), and flexible task/rule-switching (Ragozzino et al. 2003; Floresco et al. 2008; Oualian and Gisquet-Verrier 2010), to name a few. Among these hypotheses, the results from the current study may be explained most parsimoniously by the attention-based hypothesis; that is, the mPFC plays significant roles when higher-level attention is required for goal-directed response in a task (Granon and Poucet 1995; Granon et al. 1998). Prior studies showed that mPFC-lesioned rats were impaired in water-maze tasks only when more attention was required for processing partial contextual cues (Jo et al. 2007) or when starting locations were variable (thus navigational routes to a goal location were variable) from trial to trial after learning new goal locations (Granon and Poucet 1995). Similar interpretations are possible for other tasks that included fairly complex conditional relationships among stimuli (Winocur and Eskes 1998; Haddon and Killcross 2006; Lee and Solivan 2008). However, higher-level attention itself does not seem to automatically necessitate the mPFC unless the task is goal directed, because increasing the number of objects in a spontaneous object exploration task did not result in any performance deficit in mPFC-lesioned animals (Granon et al. 1996), whereas a similar study that required the rats to perform a go/no-go task for object-based scenes for obtaining reward required the mPFC (DeCoteau et al. 2009).

Due to its implicit nature of cueing in the animal's background, the VCRS task in the current study may have required more attention for processing the cueing stimuli than the VCD and TCRS tasks. Specifically, the visual stimulus in the VCD task was a target by itself (similar to a visually cued platform in the water maze) toward which the rat simply ran, and the tactile stimulus in the TCRS task was directly sensed and explicitly directed the rat to emit a specific behavior. The simple and direct associative relationships between stimulus and response in those tasks may have required less attention compared with the VCRS task. Furthermore, since each visual stimulus was presented through a single monitor in the VCD task, whereas the visual context in the VCRS task was presented through three adjacent LCD panels as a surrounding scene, the exact visual representation of the context perceived by the rat might be variable from trial to trial at the time of response selection. This may have required the rat to remain highly attentive to its background throughout a session in the VCRS task. Bussey and colleagues also reported a similar phenomenon in an object discrimination task using a touchscreen-based visual discrimination paradigm (Bussey et al. 1997); rats with mPFC lesions were only impaired when visual stimuli of the same size and luminance (thus more similar stimuli) needed to be discriminated from each other, but not when the discriminations were easier. In addition, Delatour and Gisquet-Verrier (1999) showed that rats with mPFC lesions were not impaired in a conditional task in a Y-maze using simple visual stimulus (e.g., right arm for slowly flashing light and left arm for fast flashing light). Supporting this line of reasoning, it took ∼2–3 wk for the rats to learn the VCRS task, whereas it took only 4 d on average for the rats to learn the VCD task and approximately the same amount of time for the TCRS task, suggesting that the latter two tasks were relatively easier than the VCRS task. The attention-based explanation, however, needs to be further examined with other tasks with matching attention levels (but without using visual stimuli) in the future.

In sum, the current study demonstrates the importance of the mPFC when conditional response should be made using visual context in the background to an otherwise ambiguous object. However, flexible behavioral selection was possible without normal function of the mPFC when the choice behavior was cued by elemental, but not contextual cues. The results should provide important clues for future studies to further delineating conditions in which the mPFC plays critical roles in choice behavior.

## References

[LEARNMEM-025890C1] Bussey TJ, Muir JL, Everitt BJ, Robbins TW 1997 Triple dissociation of anterior cingulate, posterior cingulate, and medial frontal cortices on visual discrimination tasks using a touchscreen testing procedure for the rat. Behav Neurosci 111: 920–936 9383514 10.1037//0735-7044.111.5.920

[LEARNMEM-025890C2] Chudasama Y, Passetti F, Rhodes SE, Lopian D, Desai A, Robbins TW 2003 Dissociable aspects of performance on the 5-choice serial reaction time task following lesions of the dorsal anterior cingulate, infralimbic and orbitofrontal cortex in the rat: Differential effects on selectivity, impulsivity and compulsivity. Behav Brain Res 146: 105–119 14643464 10.1016/j.bbr.2003.09.020

[LEARNMEM-025890C3] DeCoteau WE, McElvaine D, Smolentzov L, Kesner RP 2009 Effects of rodent prefrontal lesions on object-based, visual scene memory. Neurobiol Learn Mem 92: 552–558 19596073 10.1016/j.nlm.2009.07.003

[LEARNMEM-025890C4] Delatour B, Gisquet-Verrier P 1996 Prelimbic cortex specific lesions disrupt delayed-variable response tasks in the rat. Behav Neurosci 110: 1282–1298 8986332 10.1037//0735-7044.110.6.1282

[LEARNMEM-025890C5] Delatour B, Gisquet-Verrier P 1999 Lesions of the prelimbic-infralimbic cortices in rats do not disrupt response selection processes but induce delay-dependent deficits: Evidence for a role in working memory? Behav Neurosci 113: 941–955 10571477 10.1037//0735-7044.113.5.941

[LEARNMEM-025890C6] Floresco SB, Block AE, Tse MTL 2008 Inactivation of the medial prefrontal cortex of the rat impairs strategy set-shifting, but not reversal learning, using a novel, automated procedure. Behav Brain Res 190: 85–96 18359099 10.1016/j.bbr.2008.02.008

[LEARNMEM-025890C7] Goldman-Rakic PS 1990 Cellular and circuit basis of working memory in prefrontal cortex of nonhuman primates. Prog Brain Res 85: 325–335 2094903 10.1016/s0079-6123(08)62688-6

[LEARNMEM-025890C8] Good M, Honey RC 1991 Conditioning and contextual retrieval in hippocampal rats. Behav Neurosci 105: 499–509 1930720 10.1037//0735-7044.105.4.499

[LEARNMEM-025890C9] Granon S, Poucet B 1995 Medial prefrontal lesions in the rat and spatial navigation: Evidence for impaired planning. Behav Neurosci 109: 474–484 7662158 10.1037//0735-7044.109.3.474

[LEARNMEM-025890C10] Granon S, Vidal C, Thinus-Blanc C, Changeux JP, Poucet B 1994 Working memory, response selection, and effortful processing in rats with medial prefrontal lesions. Behav Neurosci 108: 883–891 7826511 10.1037//0735-7044.108.5.883

[LEARNMEM-025890C11] Granon S, Poucet B, Thinus-Blanc C, Changeux JP, Vidal C 1995 Nicotinic and muscarinic receptors in the rat prefrontal cortex: Differential roles in working memory, response selection and effortful processing. Psychopharmacology (Berl) 119: 139–144 7659760 10.1007/BF02246154

[LEARNMEM-025890C12] Granon S, Save E, Buhot MC, Poucet B 1996 Effortful information processing in a spontaneous spatial situation by rats with medial prefrontal lesions. Behav Brain Res 78: 147–154 8864046 10.1016/0166-4328(95)00242-1

[LEARNMEM-025890C13] Granon S, Hardouin J, Courtier A, Poucet B 1998 Evidence for the involvement of the rat prefrontal cortex in sustained attention. Q J Exp Psychol B 51: 219–233 9786783 10.1080/713932682

[LEARNMEM-025890C14] Haddon JE, Killcross S 2006 Prefrontal cortex lesions disrupt the contextual control of response conflict. J Neurosci 26: 2933–2940 16540570 10.1523/JNEUROSCI.3243-05.2006 PMC6673980

[LEARNMEM-025890C15] Hayton SJ, Lovett-Barron M, Dumont EC, Olmstead MC 2010 Target-specific encoding of response inhibition: increased contribution of AMPA to NMDA receptors at excitatory synapses in the prefrontal cortex. J Neurosci 30: 11493–11500 20739571 10.1523/JNEUROSCI.1550-10.2010 PMC4844537

[LEARNMEM-025890C16] Hirsh R 1974 The hippocampus and contextual retrieval of information from memory: A theory. Behav Biol 12: 421–444 4217626 10.1016/s0091-6773(74)92231-7

[LEARNMEM-025890C17] Horga G, Maia TV, Wang P, Wang Z, Marsh R, Peterson BS 2011 Adaptation to conflict via context-driven anticipatory signals in the dorsomedial prefrontal Cortex. J Neurosci 31: 16208–16216 22072672 10.1523/JNEUROSCI.2783-11.2011 PMC3244974

[LEARNMEM-025890C18] Jay TM, Witter MP 1991 Distribution of hippocampal CA1 and subicular efferents in the prefrontal cortex of the rat studied by means of anterograde transport of *Phaseolus vulgaris*-leucoagglutinin. J Comp Neurol 313: 574–586 1783682 10.1002/cne.903130404

[LEARNMEM-025890C31] Jo YS, Lee I 2010 Disconnection of the hippocampal–perirhinal cortical circuits severely disrupts object–place paired associative memory. J Neurosci 30: 9850–9858 20660267 10.1523/JNEUROSCI.1580-10.2010 PMC2913067

[LEARNMEM-025890C19] Jo YS, Park EH, Kim IH, Park SK, Kim H, Kim HT, Choi J-S 2007 The medial prefrontal cortex is involved in spatial memory retrieval under partial-cue conditions. J Neurosci 27: 13567–13578 18057214 10.1523/JNEUROSCI.3589-07.2007 PMC6673110

[LEARNMEM-025890C20] Kesner RP, Ragozzino ME 2003 The role of the prefrontal cortex in object-place learning: A test of the attribute specificity model. Behav Brain Res 146: 159–165 14643468 10.1016/j.bbr.2003.09.024

[LEARNMEM-025890C21] Kim JJ, Fanselow MS 1992 Modality-specific retrograde amnesia of fear. Science 256: 675–677 1585183 10.1126/science.1585183

[LEARNMEM-025890C22] Kim J, Lee I 2011 Hippocampus is necessary for spatial discrimination using distal cue-configuration. Hippocampus 21: 609–621 20623761 10.1002/hipo.20784

[LEARNMEM-025890C23] Lee I, Solivan F 2008 The roles of the medial prefrontal cortex and hippocampus in a spatial paired-association task. Learn Mem 15: 357–367 18463175 10.1101/lm.902708 PMC2364607

[LEARNMEM-025890C24] Marquis J, Kilcross S, Haddon JE 2007 Inactivation of the prelimbic, but not infralimbic, prefrontal cortex impairs the contextual control of response conflict in rats. Eur J Neurosci 25: 559–566 17284198 10.1111/j.1460-9568.2006.05295.x

[LEARNMEM-025890C25] McDonald R, Foong N, Ray C, Rizos Z, Hong N 2007 The role of medial prefrontal cortex in context-specific inhibition during reversal learning of a visual discrimination. Exp Brain Res 177: 509–519 17006687 10.1007/s00221-006-0699-9

[LEARNMEM-025890C26] Muir JL, Everitt BJ, Robbins TW 1996 The cerebral cortex of the rat and visual attentional function: dissociable effects of mediofrontal, cingulate, anterior dorsolateral, and parietal cortex lesions on a five-choice serial reaction time task. Cereb Cortex 6: 470–481 8670672 10.1093/cercor/6.3.470

[LEARNMEM-025890C27] Oualian C, Gisquet-Verrier P 2010 The differential involvement of the prelimbic and infralimbic cortices in response conflict affects behavioral flexibility in rats trained in a new automated strategy-switching task. Learn Mem 17: 654–668 21106689 10.1101/lm.1858010

[LEARNMEM-025890C28] Ragozzino ME, Kim J, Hassert D, Minniti N, Kiang C 2003 The contribution of the rat prelimbic-infralimbic areas to different forms of task switching. Behav Neurosci 117: 1054–1065 14570554 10.1037/0735-7044.117.5.1054

[LEARNMEM-025890C29] Thierry A-M, Gioanni Y, Degenetais E, Glowinski J 2000 Hippocampo-prefrontal cortex pathway: Anatomical and electrophysiological characteristics. Hippocampus 10: 411–419 10985280 10.1002/1098-1063(2000)10:4<411::AID-HIPO7>3.0.CO;2-A

[LEARNMEM-025890C30] Winocur G, Eskes G 1998 Prefrontal cortex and caudate nucleus in conditional associative learning: Dissociated effects of selective brain lesions in rats. Behav Neurosci 112: 89–101 9517818 10.1037//0735-7044.112.1.89

